# Notices, &c.

**Published:** 1866-08

**Authors:** 


### NOTICES, &c.

CHANGE.—By having a white paper cover to the JOURNAL OF HEALTH, a better quality of material and a larger amount of reading matter can be given, without any additional expense to our subscribers; hence it is hoped none will object to the change.

OUR DAUGHTERS' SCHOOLING.—The sisters Bucknall have retired from the more arduous labors of a large school for young ladies in New York city, and have removed to their beautiful country seat near New Brunswick; where, not abandoning a field altogether, in which for so many years they successfully labored, they will still continue to give instruction to a select few; this will be interesting intelligence to their patrons and scholars, which latter, after entering married life, have repeatedly come to their former teachers for the express purpose of assuring them how much they appreciated their fidelity and conscientious and untiring efforts to make their moral and literary education what it ought to be, and which they more highly valued now, than when they stood in the relation of pupils and teachers. This simple fact of itself tells volumes in just praise of these admirable and able instructors of so many of the daughters of New York.

Being in the midst of a farming region of great fertility, the necessary expenses of boarding are lessened, while its known salubrity, the social surroundings, the many churches, the flourishing Theological Seminary, and the accessibility to New York and Philadelphia by Rail, many times a day, all make New Brunswick one of the very best locations in the Union for the proper education of our daughters.

THE AMERICAN TRACT SOCIETY, 150 Nassau St., New York, have issued "Leaves of Life," being striking facts illustrative of select passages from the Bible; a class of books which ought to be multiplied by millions and thrown broadcast over the land; for whatever tends to impress the minds of the young as to the meaning of Scripture, its truth, and its divine origin, helps to benefit and bless the race; and thrice happy are they individually, who can lean trustingly upon every Bible statement, and feed upon it, and feel assured always that "it is a faithful saying and worthy of all acceptation." —Of a similar nature is "Food for Lambs."

An instructive narration for the young is "Lyttonville; or, the Irish Boy in Canada;"—showing how beautiful it is to return good for evil.

Messrs. J. G. Broughton and Wyman of the American Tract Society, 13 Bible House, New York, and 28 Cornhill, Boston, have issued "A Word to Sabbath-School Teachers," urging them to more diligent attention to their work; to throw into it more prayer and a more intense looking for present results. ——"Ten Helps to Joy and Peace;" also, "Bible Sketches, and their Teachings.

THE CROSS IN THE CELL.—In regard to this book, by Rev. N. Adams, D. D., of Boston, which is designed as a guide to inquirers, an eminent divine and experienced pastor says:

"I am filled with sincere delight by the book. I know of no work uninspired in which the gospel is preached more skillfully, plainly, and affectionately. It is 'Baxter's Call,' 'James's Anxious Inquirer,' etc., only better than any of them. It is the best book I know of to put into the hands of any one—judge or culprit, old or young—when one wishes to teach 'more perfectly in this way.' It has all the charm of a story and all the power of the pulpit."

—Last evening a lady admirer of the JOURNAL forwarded the following scrap from an unnamed newspaper, and this morning another came from California.

"SLEEPING TOGETHER.—Hall's *Journal of Health*, which claims to be the highest authority in medical science, has taken a stand against married people sleeping together, and thinks they had better sleep in adjoining rooms. It says kings and queens never sleep together, and why should other people? Think of seperating a newly married pair on a cold winter's night, because Hall's *Journal of Health* said so!

"We suppose the reason that Mr. Hall has taken the stand that he has, is because he has studied the science of medicine so much in his young days, as to become round shouldered, and so much deformed otherwise as to prevent the fair sex from admiring him very much. If he is not deformed in his body, he certainly is in his head. Just think of it! Our wife going to bed in one room and us in another, especially when the rats are as bad as they are at our house. Mr. Hall can just go any where he pleases, provided he has the wherewith to pay his fare, but we'll snooze with our wife as long as we've got one.

"A good many people sleep together in these parts, who ought not even to sleep in adjoining rooms; but Hall's Jonrnal of Health is'nt sufficiently popular to break up the practice."

To all of which we have only to say, we are straight as a ramrod, as lively as a cricket and as brisk as a bee; our poll is as black as a big tom-cat in the dark; we have no bricks in the hat but have some rocks in the pocket; and as to this sleeping business, we think we are in the right. What's the use of having one wife or a husband, if you are fast asleep? We do not object to the idiosyncrosies of our critical young friend; this is a free country, but as for ourselves, we rather prefer being wide awake when things are going on, and if any one can sleep under the circumstances it is because he's "no account."

